# CNPY2 in Solid Tumors: Mechanisms, Biomarker Potential, and Therapeutic Implications

**DOI:** 10.3390/biology14020214

**Published:** 2025-02-18

**Authors:** Sayan Mullick Chowdhury, Feng Hong, Christian Rolfo, Zihai Li, Kai He, Robert Wesolowski, Amir Mortazavi, Lingbin Meng

**Affiliations:** 1 Division of Medical Oncology, Department of Internal Medicine, The Ohio State University Comprehensive Cancer Center, Columbus, OH 43210, USA; sayan.mullickchowdhury@osumc.edu (S.M.C.); feng.hong@osumc.edu (F.H.); christian.rolfo@osumc.edu (C.R.); zihai.li@osumc.edu (Z.L.); kai.he@osumc.edu (K.H.); robert.wesolowski@osumc.edu (R.W.); amir.mortazavi@osumc.edu (A.M.); 2 Pelotonia Institute for Immuno-Oncology, The Ohio State University Comprehensive Cancer Center, Columbus, OH 43210, USA

**Keywords:** canopy FGF signaling regulator 2, biomarker, therapeutic target, angiogenesis, oncogenesis, immunotherapy, tumor microenvironment

## Abstract

Canopy FGF signaling regulator 2 (CNPY2) has emerged as a crucial player in cancer development by promoting cell proliferation, tissue repair, and angiogenesis. This review synthesizes the current understanding of CNPY2’s role in solid tumors, particularly renal cell carcinoma, prostate cancer, hepatocellular carcinoma, and non-small-cell lung cancer. CNPY2 modulates key pathways such as p53, MYLIP, NF-κB, and AKT/GSK3β, thereby driving tumor growth and progression. In renal cell carcinoma, CNPY2 paradoxically promotes tumor growth through p53 upregulation, while in hepatocellular carcinoma, CNPY2 drives cell cycle progression via p53 destabilization. In prostate cancer, it enhances tumor progression by stabilizing androgen receptors through MYLIP interaction, and in non-small-cell lung cancer, it contributes to chemoresistance and metastasis through NF-κB and AKT/GSK3β signaling. Additionally, CNPY2 influences the tumor microenvironment, impacting immune function and metastatic potential. As a potential biomarker, CNPY2 shows promise for cancer detection and prognosis, particularly when used in combination with other markers. Early therapeutic strategies, including siRNA and miRNA approaches, are under exploration, though challenges remain due to CNPY2’s expression in normal tissues and potential off-target effects. This review underscores the need for further research to fully elucidate CNPY2’s oncogenic mechanisms and develop targeted therapies. Improved understanding of CNPY2’s diverse roles may lead to novel diagnostic and therapeutic approaches in solid tumors.

## Simple Summary

This review examines a protein called CNPY2, which is found in higher amounts in many types of cancer such as those affecting the kidney, liver, lung, and prostate. The study aimed to understand how this protein contributes to cancer by helping tumors grow, spread, and resist treatment, and to explore whether it can serve as a useful marker for early detection or a target for new therapies. Researchers found that CNPY2 is involved in several processes that make cancer cells more aggressive and better able to avoid the body’s natural defenses. These findings suggest that by tracking or targeting CNPY2, doctors might be able to diagnose cancers more accurately and develop treatments that are more effective and personalized. In short, understanding the role of CNPY2 could lead to improved care for patients by offering new ways to slow down or stop tumor growth, ultimately reducing the impact of cancer on society.

## 1. Introduction

Canopy FGF signaling regulator 2 (CNPY2) has emerged as a critical player in cancer biology, with increasing evidence pointing to its pivotal role in tumor development and progression [1,2,3]. As a member of the CNPY family of proteins, CNPY2 is an endoplasmic reticulum (ER) luminal protein that has garnered significant attention due to its multifaceted functions in cell signaling, tissue repair, and angiogenesis [4,5]. Dr. Feng Hong and colleagues first identified CNPY2 as a key regulator of the unfolded protein response (UPR), demonstrating its role in activating the PERK–CHOP pathway to mediate cellular stress responses [4]. Recent studies have unveiled CNPY2’s involvement in various oncogenic processes, including cell proliferation, angiogenesis, and metastasis [2,5,6]. The protein’s expression is directly regulated by hypoxia-inducible factor 1-alpha (HIF-1α), linking it to hypoxic conditions often found in rapidly growing tumors [5]. Moreover, Hong et al. showed that CNPY2 modulates key pathways such as p53 signaling, potentially inhibiting cell death and promoting cancer cell survival [7].

The significance of CNPY2 in cancer biology is underscored by its overexpression across various solid tumors, including renal cell carcinoma (RCC) [8], prostate cancer [3], and hepatocellular carcinoma (HCC) [7]. This dysregulation appears to be an early event in carcinogenesis, suggesting its potential role as both a diagnostic marker and therapeutic target. Each cancer type appears to utilize CNPY2 through distinct mechanisms, highlighting the protein’s versatility in promoting oncogenesis. For instance, in prostate cancer, CNPY2 has been found to stabilize androgen receptors [3], while in hepatocellular carcinoma, it promotes cell cycle progression through p53 destabilization [7]. These tissue-specific mechanisms suggest that CNPY2 may have evolved to serve context-dependent functions in different cellular environments.

As research into CNPY2’s role in cancer has expanded rapidly in recent years, there is a pressing need to synthesize and critically evaluate the current state of knowledge. This review aims to provide a comprehensive overview of CNPY2’s functions in solid tumors, with a particular focus on its mechanisms of action, its impact on the tumor microenvironment, and its potential as a biomarker and therapeutic target. We begin by examining the structure and function of CNPY2, its diverse roles in specific solid tumors, and its influence on the tumor microenvironment. Additionally, we will discuss the emerging potential of CNPY2 as a biomarker for cancer detection and prognosis, as well as early efforts to target CNPY2 therapeutically. Finally, we highlight critical knowledge gaps and future research directions that could accelerate the translation of CNPY2-based interventions into clinical practice. By consolidating the latest findings and identifying key areas for future research, this review seeks to foster a deeper understanding of CNPY2’s significance in cancer biology and its potential implications for cancer diagnosis and treatment.

## 2. CNPY2 Structure and Function

**Figure 1:**
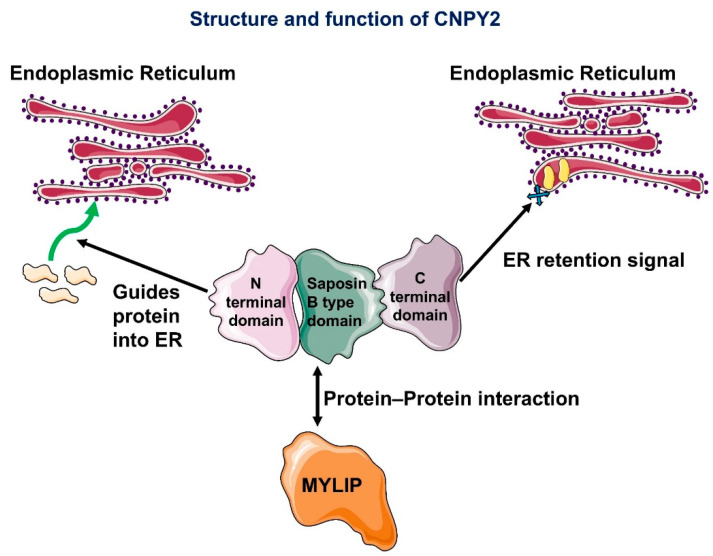
Schematic representation of the CNPY2 protein domains. The N-terminal domain (signal peptide) directs CNPY2 into the endoplasmic reticulum (ER), the saposin B-type domain mediates protein–protein interactions (notably binding with MYLIP), and the C-terminal domain contains the ER retention signal that keeps CNPY2 localized to the ER.

The CNPY family is composed of the CNPY1, CNPY2, CNPY3, CNPY4, and CNPY5 proteins, all of which play important roles in growth and development [5,9,10,11]. All members of the CNPY family exhibit a similar domain structure, comprising an N-terminal domain with a consensus signal peptide, a saposin B-type domain for protein interactions, and a C-terminal domain with an ER retrieval sequence (Figure 1), enabling their active participation in cell signaling pathways [4,12]. CNPY1 is known to act as a positive feedback regulator for FGF and plays a significant role in brain development, as shown in zebrafish studies [13]. Similarly, CNPY2 is thought to contribute to brain development [14]. Structurally, CNPY2 contains a saposin B-type domain that can be modulated by FGF21 [15]. It also has a hypoxia-responsive element in its promoter region, which likely responds to hypoxic environments by upregulating CNPY2 expression [5]. At the molecular level, the CNPY2 gene codes for a peptide that includes both the saposin B-type domain and a sequence for ER retention. Overall, very limited data are currently available on CNPY2’s structure and function [14]. A 2003 article demonstrated that CNPY2 interacts with myosin-regulatory light chain-interacting protein (MYLIP), an E3 ubiquitin ligase that marks targets for degradation in lysosomes [16]. CNPY2 protects certain targets, such as very-low-density lipoprotein receptor (VLDLR), apolipoprotein E receptor 2 (ApoER2), and androgen receptors (ARs), from lysosomal degradation through MYLIP-mediated ubiquitination [17]. CNPY2 has also been reported to regulate the expression of low-density lipoprotein receptor and FGF21 [15]. In small animal studies, CNPY2 expression was found to vary across different organs, suggesting that CNPY2 likely has a wide variety of functions that remain to be fully explored [14].

## 3. Mechanisms of CNPY2 Action in Cancer

**Figure 2:**
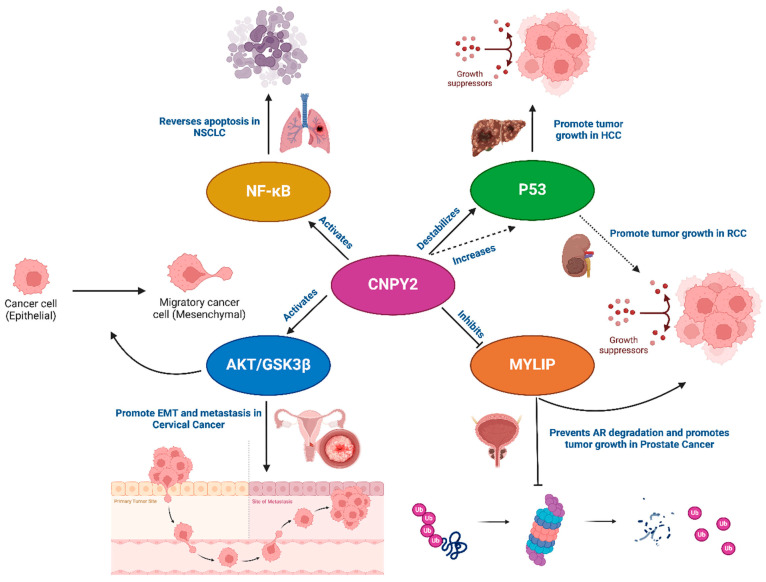
Overview of CNPY2’s multifunctional roles in solid tumors (created with BioRender.com). A schematic illustrating how CNPY2 interacts with multiple molecular pathways—p53, MYLIP, NF-κB, and AKT/GSK3β—to drive distinct oncogenic processes across different cancer types.

The role of CNPY2 in cancer growth is thought to be mediated through various mechanisms, which can differ depending on the type of cancer. Primarily, CNPY2 has been shown to influence angiogenesis, thereby regulating cancer cell growth, proliferation, and metastasis [5,18]. Angiogenesis is a crucial component of cancer growth, as it provides the necessary blood supply for tumors to expand and metastasize [18,19,20]. Growth factors that directly affect cancer angiogenesis are often key contributors to oncogenesis [21,22,23]. Additionally, CNPY2 overexpression has been found to inhibit cell death, or apoptosis, by suppressing the tumor suppressor gene p53 [7]. Since p53 normally works to prevent uncontrolled cell growth, its inhibition by CNPY2 can lead to unchecked tumor development [24]. CNPY2’s influence on oncogenesis appears to involve several key signaling pathways. One of the mechanisms is believed to involve the FGF21 pathway, though the specific details of this interaction remain unclear [15]. A 2017 study showed that CNPY2 is released during ER stress, which activates the unfolded protein response, potentially improving cancer cell survival under these stressful conditions [4]. In vitro experiments also revealed that CNPY2 overexpression in non-small-cell lung cancer (NSCLC) cells could reverse cisplatin-induced apoptosis by activating the NF-κB pathway [25]. Furthermore, CNPY2 overexpression was shown to enhance the invasive and metastatic capabilities of these cancer cells [12]. This process was mediated through the AKT/GSK3β pathway, ultimately leading to reduced E-cadherin expression and promoting epithelial–mesenchymal transition (EMT). A similar activation of the AKT pathway has also been observed in cervical cancer cells that overexpress CNPY2 [26]. In prostate cancer, CNPY2 promotes tumor growth by interacting with MYLIP, preventing the ubiquitination and degradation of androgen receptors (ARs) through the ubiquitin–proteasome pathway [3]. In RCC, CNPY2 upregulation paradoxically leads to increased p53 expression, which in this case promotes RCC growth rather than inhibiting it [8]. In HCC, CNPY2 is significantly upregulated, and this upregulation is associated with poorer survival. It has been demonstrated that increased CNPY2 expression in HCC destabilizes p53, driving cell cycle progression and leading to uncontrolled tumor growth [27]. As summarized in Figure 2, these examples illustrate that CNPY2 plays a role in multiple pathways across different cancers, each of which may rely on unique mechanisms to promote oncogenesis. Understanding these diverse pathways further highlights CNPY2’s potential as a key regulator of cancer progression.

## 4. CNPY2 in Specific Solid Tumors

### 4.1. Renal Cell Carcinoma

RCC is the most prevalent type of kidney cancer in humans [28,29,30], and studies have shown that RCC cells frequently overexpress CNPY2 [8]. A 2017 study demonstrated that high levels of CNPY2 expression are directly correlated with the growth and progression of human RCC cells [8]. Interestingly, this mechanism was found to involve the upregulation of TP53 expression, which is counterintuitive, as TP53 is typically known as a tumor suppressor, and its upregulation would generally inhibit cell growth [31,32]. However, in RCC cell lines, TP53 upregulation correlated with improved survival of RCC cells. CNPY2 knockdown significantly inhibited the growth of RCC cells by approximately 50%, with corresponding downregulation of TP53 [8]. In addition, CNPY2 downregulation also reduced TP53 expression and decreased p53-mediated activation of the CDKN1A gene in RCC cells [8].

This seemingly paradoxical effect of p53 promoting cancer cell survival rather than suppressing growth can be understood in the context of p53’s complex roles in cancer. Despite p53 being widely recognized for its tumor-suppressive role through inducing cell cycle arrest or apoptosis [33,34], certain cancer contexts reveal “gain-of-function” or mutant p53 activities that instead promote tumorigenesis [35,36,37]. In RCC, it is possible that the observed increase in p53 reflects either noncanonical p53 functions or the presence of p53 variants that drive proliferation and metabolic adaptation. Some RCC models have suggested that wild-type p53 can interact with altered cofactors or signaling modules (such as HIF pathways) that reprogram its function from growth inhibition to supporting cell survival [38,39]. Additionally, certain p53 post-translational modifications, such as phosphorylation and acetylation, can shift its downstream target genes from pro-apoptotic to pro-proliferative in a subset of tumors [24,40,41]. Other cancers, such as certain breast and ovarian tumors, also demonstrate aberrant or paradoxical p53 activities [42,43,44]. This phenomenon underscores the importance of tumor-specific contexts in determining whether p53 exerts anticancer or pro-survival effects. Future studies clarifying whether RCC cells primarily express mutant p53 forms or leverage unique transcriptional coactivators may help explain this paradoxical role of p53 in RCC.

### 4.2. Prostate Cancer

CNPY2 has also been found to play a role in prostate cancer progression through its modulation of the androgen receptor (AR) [3]. It is well-established that AR overexpression and signaling pathways mediated by ARs are critical for the survival of prostate cancer cells [45,46,47]. In *Drosophila* models, CNPY2 expression was shown to promote the growth, proliferation, and metastasis of prostate cancer cells [48]. A 2018 study demonstrated that AR can be targeted for degradation through ubiquitination mediated by MYLIP, an E3 ubiquitin ligase [3]. However, the study found that increased CNPY2 expression inhibits this process by interacting with and suppressing MYLIP’s function. This inhibition prevents AR degradation, resulting in elevated AR levels in prostate cancer cells and promoting their survival through AR-mediated signaling.

The binding between CNPY2 and MYLIP likely involves specific domain–domain interactions, particularly within the saposin B-type domain of CNPY2 and the catalytic RING domain of MYLIP. By preventing MYLIP from tagging AR for lysosomal or proteasomal degradation, CNPY2 enhances AR stability and sustains downstream transcriptional programs essential for prostate tumor growth [3]. Understanding these molecular details is crucial, as targeted disruption of the CNPY2-MYLIP interface could effectively reduce AR levels in prostate cancer cells. Moreover, it remains to be studied whether hypoxia-driven upregulation of CNPY2 (via HIF-1α) additionally modifies any of these post-translational events, creating a feedforward loop that further stabilizes AR in castration-resistant prostate cancer.

This potential feedforward mechanism is particularly relevant because it is well known that androgen deprivation through castration can create a hypoxic environment in prostate cancer, leading to increased expression of HIF-1α [49,50,51,52], which in turn may activate CNPY2 expression [26,53]. Based on this, it is hypothesized that hypoxia induced by castration may drive the overexpression of CNPY2 observed in castration-resistant prostate cancer. This raises the possibility that targeting CNPY2 could be a promising therapeutic approach in castration-resistant prostate cancer, although further research is needed to fully explore this idea.

### 4.3. Hepatocellular Carcinoma

CNPY2 has been found to be significantly elevated in HCC tissues and cell lines [2,7,27]. Gene expression profile surveys have previously reported that CNPY2 overexpression in HCC cells correlates with increased metastasis in primary HCC cell lines [2]. A 2018 study initially identified CNPY2 as playing a pivotal role in HCC progression and cell survival [2]. In a 2021 study, the knockdown of CNPY2 in HCC cell lines significantly decreased both cell survival and invasive potential [27]. Conversely, transfection of CNPY2-containing plasmids into the same cell lines led to increased CNPY2 expression, enhancing the survival capacity of HCC cells. This study confirmed the involvement of CNPY2 in the development and progression of HCC. A 2022 study further supported these findings, delving deeper into the molecular mechanisms involved [7]. The study demonstrated that CNPY2 deletion protects against diethylnitrosamine-induced HCC in a mouse model, and analysis of human HCC samples revealed that higher CNPY2 expression is associated with worse survival outcomes [7]. Mechanistic studies revealed that CNPY2 upregulation led to p53 dysregulation through the unfolded protein response, promoting cell cycle progression and increased HCC survival [7]. Transcriptome analysis also showed that CNPY2 regulates the expression of other oncogenes, such as c-Jun and FGF21 [7]. These findings suggest that CNPY2 plays a crucial role in HCC development and progression. Given this, further investigation into the potential of CNPY2 as a therapeutic target for HCC treatment is warranted. Additionally, CNPY2 could be explored as a prognostic marker in HCC.

### 4.4. Non-Small-Cell Lung Cancer

CNPY2 plays multiple critical roles in NSCLC progression through distinct molecular pathways. Analysis of TCGA data has shown that CNPY2 expression is significantly increased in both adenocarcinoma and squamous cell carcinoma, which together account for approximately 85% of NSCLC cases [12]. The elevated CNPY2 expression correlates with poor survival outcomes in NSCLC patients [25,54].

CNPY2’s oncogenic functions in NSCLC operate through several key mechanisms. First, it promotes resistance to chemotherapy by inhibiting apoptosis via NF-κB pathway activation. Studies have shown that CNPY2 overexpression can counteract cisplatin-induced apoptosis by activating the NF-κB signaling pathway, which in turn promotes the expression of anti-apoptotic factors such as c-IAP1, c-IAP2, XIAP, Bcl-2, and Bcl-xl [25]. This NF-κB-mediated chemoresistance mechanism appears to be unique to NSCLC cells and has not been observed in other cancer types. Second, CNPY2 drives NSCLC metastasis by promoting EMT through the AKT/GSK3β pathway. CNPY2 overexpression activates AKT, leading to GSK3β phosphorylation and inactivation. This results in increased Snail expression and subsequent downregulation of E-cadherin, ultimately enhancing the invasive and metastatic capabilities of NSCLC cells [12]. The importance of this pathway has been demonstrated through rescue experiments where the inhibition of AKT signaling prevents the malignant transformation of cells with upregulated CNPY2.

Regulation of CNPY2 expression in NSCLC has been linked to microRNA-mediated control. Specifically, miR-30a-3p has been identified as a direct negative regulator of CNPY2 [54]. miR-30a-3p expression is significantly decreased in NSCLC tissues and cell lines, contributing to CNPY2 upregulation. Experimental restoration of miR-30a-3p levels through mimic treatment results in reduced CNPY2 expression and subsequent suppression of NSCLC cell growth, invasion, and migration, suggesting therapeutic potential.

These findings highlight CNPY2 as a multifaceted oncogenic driver in NSCLC, affecting both chemoresistance and metastatic potential through distinct molecular pathways. The identification of miR-30a-3p as a CNPY2 regulator provides a potential therapeutic strategy for targeting CNPY2 in NSCLC treatment.

### 4.5. Other Solid Tumors

CNPY2’s role in other cancers, including cervical cancer, colorectal cancer, gastric cancer, and esophageal squamous cell carcinoma (ESCC), has also been explored. In cervical cancer cells, CNPY2 upregulation has been linked to a worse prognosis [26]. Experimental validation through CNPY2 knockdown demonstrated reduced cancer cell survival, confirming its critical role in disease progression [26]. Mechanistically, CNPY2 overexpression enhances cellular bioenergetics by increasing oxygen consumption, ATP production, and glucose uptake. These metabolic changes are orchestrated through HIF-1α signaling, which functions as an upstream regulator of CNPY2 expression [25,26].

In colorectal cancer, elevated levels of CNPY2 expression were observed in cancerous tissues when compared to healthy controls [1,54]. The same study showed that CNPY2 knockdown increased p53 activity in colorectal cancer cell lines, suggesting that CNPY2 may play an important role in the development and progression of colorectal cancer [1]. Another 2017 study found elevated CNPY2 expression in colorectal cancer cells compared to normal colonic tissue and epithelium. Interestingly, survival analysis revealed that patients with low CNPY2 expression had worse 5-year overall survival compared to those with higher expression [6]. A 2018 study further evaluated the efficacy of CNPY2 as a biomarker in colorectal cancer, finding that combining CNPY2 with CEA and CA19-9 significantly improved diagnostic accuracy compared to using each marker individually [55]. The study concluded that the diagnostic efficacy of CEA, CA19-9, and CNPY2 taken together were far superior compared to each of these markers alone in the detection of colorectal cancer [55].

In gastric cancer, CNPY2 expression is regulated via the miR-545-5p axis, influenced by the long non-coding RNA LINC00342, which is overexpressed in GC. This regulatory mechanism suggests a potential oncogenic role for CNPY2, though further studies are needed to clarify its precise contribution to gastric tumorigenesis [56].

In ESCC, mass spectrometry studies identified CNPY2 as an interacting partner of Ezrin, a protein that links the plasma membrane to the cytoskeleton and plays a key role in ESCC progression. While the functional significance of this interaction remains unexplored, high CNPY2 expression has been associated with lower overall survival and disease-free survival in ESCC patients. The study also found that an Ezrin-interacting protein signature could be used to predict tumor recurrence and survival outcomes, highlighting CNPY2’s potential role in ESCC progression [57].

Overall, it is clear that CNPY2 is upregulated in various cancers and may contribute to tumorigenesis through different mechanisms. While some cancers may share common pathways for CNPY2-driven tumor initiation and proliferation, others appear to utilize distinct mechanisms. More detailed studies are needed to fully elucidate the molecular mechanisms of CNPY2 in different cancers.

## 5. CNPY2 and Tumor Microenvironment

Recent research has conclusively demonstrated that the tumor microenvironment plays a critical role in tumor development, proliferation, and metastasis [58,59,60]. The tumor microenvironment, or tumor milieu, refers to the ecosystem within which the tumor grows and interacts with surrounding cells and molecules [60]. These interactions can significantly influence tumor growth, cellular communication, and response to therapeutic agents. Additionally, the tumor microenvironment serves as a site where tumor cells react to external molecules and release their contents upon undergoing apoptosis.

One important aspect of the tumor microenvironment in cancer is the induction of ER stress, a process triggered by the accumulation of misfolded proteins that can inadvertently promote tumor growth [61,62]. Recent findings indicate that CNPY2 is upregulated in response to ER stress, driving pro-survival pathways in cancer cells [4,63]. A 2018 study in HCC cells showed that CNPY2 interacts directly with the tumor microenvironment, including various immune cells, such as T cells, as well as extracellular proteins and cytokines that contribute to HCC proliferation [2]. Notably, knocking out CNPY2 in HCC cell lines reduced tumor hemorrhage, which typically protects tumors from immune system attacks, underscoring CNPY2’s role in linking ER-stress-mediated survival responses to tumor microenvironment modulation [2].

Beyond its metabolic functions, CNPY2 plays a critical role in immune regulation within the tumor microenvironment. T lymphocytes, a crucial component of cancer immunology, are often rendered dysfunctional in the tumor microenvironment due to hostile conditions and continuous antigen stimulation [64,65]. Such stress on T cells has been linked to the initiation of ER stress, resulting in CNPY2 upregulation and subsequent T cell dysfunction through the unfolded protein response [66]. By contributing to maladaptive unfolded protein response signaling, CNPY2 could serve as a novel checkpoint in T cell biology within the tumor microenvironment. Elevated CNPY2 expression may exacerbate T cell dysfunction, either by promoting pro-survival yet functionally “exhausted” phenotypes or by driving immunosuppressive signals. T cells experiencing chronic ER stress often upregulate inhibitory receptors, lose cytotoxic function, and produce fewer effector cytokines, enabling tumor cells to evade immune-mediated killing [67,68,69,70]. Therapeutically targeting CNPY2 in T cells, in conjunction with existing checkpoint inhibitors (e.g., anti-PD-1, anti-CTLA-4), might help restore robust antitumor immunity.

CNPY2 expression is also influenced by hypoxia, a condition common in tumor microenvironments. For example, in cervical cancer cells, CNPY2 upregulation in response to hypoxic stress has been shown to promote cancer cell survival by upregulating glycolysis [26]. Studies have shown that CNPY2 can enhance angiogenesis and stimulate the proliferation and growth of smooth muscle cells under hypoxic conditions within the tumor microenvironment [2,5]. Additionally, CNPY2-induced upregulation of survival pathways can stabilize HIF-1α under hypoxia, potentially creating a positive feedback loop where HIF-1α both triggers and benefits from CNPY2 expression [5]. This synergy can lead to enhanced vascular density and a more aggressive tumor phenotype. Mechanistically, CNPY2 has been found to activate targets such as CDC42, PAK1, and FAK in smooth muscle cells under hypoxic stress [2,66]. In small animal studies, CNPY2 expression significantly promoted revascularization in the mouse retina following ischemic reperfusion injury, further highlighting its role in promoting tissue repair and vascular growth under stress conditions [2,66].

Although direct evidence is currently lacking, it is plausible that CNPY2 also modulates other stromal components, including cancer-associated fibroblasts (CAFs). CAFs can secrete extracellular matrix proteins, growth factors, and cytokines that profoundly influence tumor progression. Given CNPY2’s role in regulating ER stress and hypoxia responses, one can hypothesize that its expression in CAFs—if elevated—could reshape the stromal “niche”, fostering further tumor invasiveness and immune suppression. Future studies in co-culture models or patient-derived xenografts may help clarify whether CNPY2 expression in stromal cells mirrors or enhances the pro-tumoral roles observed in tumor cells themselves.

## 6. CNPY2 as a Biomarker and Therapeutic Target

Given that CNPY2 is overexpressed in various cancers [2,3,56], it has strong potential as a biomarker for both diagnosis and prognosis. For instance, a 2018 study assessed the effectiveness of CNPY2 as a biomarker for detecting colorectal cancer [55]. The study concluded that the combined diagnostic efficacy of CEA, CA19-9, and CNPY2 was significantly higher compared to using each of these markers individually (*p* < 0.0167). Another 2017 study involving colorectal cancer patients showed that lower CNPY2 expression (specifically isoform 2) was associated with poorer survival [6]. In multivariable analyses, CNPY2 isoform 2 was identified as a predictor of 5-year overall survival in both the training and validation cohorts, indicating its potential as a prognostic marker in colorectal cancer.

While CNPY2’s presence in blood samples has been confirmed, including in healthy individuals and cancer patients, further investigation of its utility in liquid biopsy applications could enhance its clinical value. Studies have shown that ER-stress-related proteins can be secreted in exosomes or microvesicles [71,72], suggesting potential mechanisms for CNPY2’s presence in circulation. Large-scale prospective trials are now needed to fully characterize circulating CNPY2 levels across various solid tumors and evaluate its potential for monitoring tumor progression or therapy response via non-invasive sampling.

Building on these findings, despite encouraging results in colorectal cancer, a more systematic comparison of CNPY2 with well-known clinical biomarkers could shed light on its broader utility. For example, prostate cancer diagnosis frequently relies on prostate-specific antigen (PSA) levels [73,74], and hepatocellular carcinoma (HCC) often employs alpha-fetoprotein (AFP) screening [75,76]. Pancreatic cancer utilizes CA19-9 [77], and breast cancer can involve markers like CA15-3 [78]. Constructing comparative studies that evaluate the sensitivity, specificity, and predictive values of CNPY2 against these established biomarkers could provide valuable insights.

Beyond its diagnostic potential, targeting CNPY2 for cancer therapy can involve either directly inhibiting CNPY2 or focusing on downstream effectors. For example, MYLIP, an E3 ubiquitin kinase, is a key target of CNPY2 and is implicated in the development of several cancers [3,79]. MYLIP could be a viable therapeutic target in diseases like prostate cancer, where CNPY2 is involved [3]. The inhibition of CNPY2 expression has already shown promise as a therapeutic strategy in various in vitro and in vivo studies. Approaches to knock down CNPY2 include the use of siRNA to inhibit CNPY2 in HCC cells [7], as well as miR-30a-3p, a potential therapeutic option to downregulate CNPY2 in NSCLC cells [54]. The use of miR-30a-3p resulted in decreased growth, invasion, and migration of NSCLC cells. There is substantial potential for the development of cancer therapies targeting CNPY2 or its downstream effectors, and further exploration in this area is highly warranted. Although current preclinical data are promising, including both in vitro and in vivo studies, no clinical trials targeting CNPY2 inhibition are currently underway as a cancer treatment strategy. Continued research could eventually lead to the development of clinical therapies that specifically target CNPY2.

## 7. Challenges and Future Perspectives

A significant challenge in studying CNPY2 in solid tumors is its widespread expression in normal tissues, not just in cancerous ones [80]. This lack of specificity complicates its use as a reliable biomarker for cancer detection or prognosis. Additionally, targeting CNPY2 for therapeutic downregulation poses potential risks, as it plays vital roles in key physiological processes, including cellular stress response, angiogenesis, and smooth muscle cell proliferation. Non-specific inhibition in healthy tissues may lead to unintended side effects. Although detailed toxicology studies on CNPY2-targeted therapies are currently lacking, it is reasonable to assume that off-target effects could resemble those of other anti-angiogenic agents, including impaired wound healing, bleeding complications due to disrupted vascular growth, and potential organ damage resulting from compromised stress response and angiogenesis.

Recent data from Dr. Hong Feng’s lab have offered valuable insight into the potential therapeutic window for CNPY2 targeting. Whole-body knockout (KO) of CNPY2 in mice is non-lethal, with KO mice being slightly smaller than their wild-type (WT) counterparts and exhibiting normal development [4], suggesting that CNPY2 may not be critical for normal tissue development or that its function could be compensated by other CNPY family proteins. This finding contrasts sharply with in vitro and in vivo evidence that CNPY2 is critical for the growth of certain cancers, including HCC and RCC [8,27]. Taken together, these observations imply that blocking CNPY2 might selectively impair tumor growth without causing severe systemic toxicity, thereby strengthening the rationale for investigating CNPY2 as a therapeutic target.

Although whole-body CNPY2 KO mice appear viable and largely normal, caution is still warranted in translating these observations to humans. CNPY2 may perform supportive or compensatory roles in certain tissues that only become evident under physiological stress or in advanced age. Moreover, systemic inhibition in cancer patients could intersect with comorbidities or medications that were not modeled in KO mice. Techniques such as tumor-specific promoters or antibody–drug conjugates may help direct CNPY2-targeted treatments to malignant tissues, reducing the likelihood of off-target toxicities. Rigorous preclinical toxicology studies in diverse animal models, including those that mimic human comorbidities, will be essential for advancing CNPY2 inhibitors or knockdown strategies into clinical trials.

Despite these challenges, emerging technologies offer promising directions that could unlock the full therapeutic potential of CNPY2. One promising avenue is to explore more deeply the immunological aspects of CNPY2, particularly how it may modulate T cell dysfunction or promote immune escape within the tumor microenvironment. Single-cell RNA sequencing and proteomic analyses could reveal whether CNPY2 knockout or knockdown might reinvigorate antitumor T cell responses or synergize with checkpoint inhibitors. Refining the delivery of siRNA or miRNA remains another key objective, given the encouraging in vitro data but unresolved challenges in achieving stable and tumor-specific uptake. Nanoparticle formulations, exosome-based vehicles, or tissue-specific promoters may offer strategies to overcome these limitations while minimizing off-target effects. Finally, the potential synergy between CNPY2 inhibitors and novel anticancer modalities—ranging from oncolytic virotherapies to next-generation targeted agents—warrants systematic screening in patient-derived xenografts or organoids. By clarifying which tumor types are most dependent on CNPY2 and identifying optimal combination regimens, researchers can better prioritize clinical translation of CNPY2-targeting therapeutics.

## 8. Conclusions

In the past decade, research into CNPY2 has significantly advanced our understanding of its role in various diseases, particularly cancer. The findings position CNPY2 as an important player in cancer biology, especially in solid tumors, and highlight its potential as both a therapeutic target and biomarker. This review has synthesized current knowledge of CNPY2’s involvement in multiple oncogenic pathways, including its interactions with p53, MYLIP-mediated androgen receptor regulation, NF-κB-dependent chemoresistance, AKT/GSK3β-mediated metastasis, and its complex roles in the tumor microenvironment involving immune modulation, hypoxic responses, and angiogenesis.

Despite considerable promise, several hurdles remain. CNPY2 is expressed in normal tissues, making the therapeutic window narrow. Conflicting data in certain cancer models further complicate the picture, emphasizing the need for large-scale, multi-omics studies to clarify the contexts in which CNPY2 serves as a decisive driver of tumorigenesis. Nonetheless, the advent of targeted delivery technologies, combined with deeper exploration of CNPY2’s immunological impact, could lead to groundbreaking therapies that capitalize on CNPY2 inhibition while minimizing harmful off-target effects. With continued innovation, it is conceivable that CNPY2-directed interventions will progress from early research stages to clinical testing, ultimately offering new hope for patients battling cancers that rely on this key regulator.

## Data Availability

Not applicable.
